# Intravitreal Ziv-Aflibercept in Treatment of Naïve Chronic Central Serous Chorioretinopathy Related Choroidal Neovascular Membrane

**DOI:** 10.1155/2017/5036248

**Published:** 2017-11-23

**Authors:** Nishant Radke, Charudutt Kalamkar, Amrita Mukherjee, Snehal Radke

**Affiliations:** Shri Ganesh Vinayak Eye Hospital, Raipur, India

## Abstract

*Purpose. *To study the effect and outcome of intravitreal Ziv-Aflibercept (IVZ) in treatment of Chronic Central Serous Chorioretinopathy (CSCR) related Choroidal Neovascular Membrane (CNVM).* Methods. *A case report of 48-year-old male patient treated with 1.25 mg/0.05 ml IVZ (total 3 doses at monthly intervals) in CSCR related CNVM. Pre- and posttreatment fundus fluorescein angiography (FFA) and Optical Coherence Tomography (OCT) were done to document response along with improvement in visual acuity.* Patients. *Single eye of a 48-year-old male patient.* Results. *Regression of CNVM was noted with improvement of macular contour and thickness on OCT and cessation of leakage on FFA. Visual acuity improved from 3/60, <N36 to 6/12, N12.* Discussion.* Anti-VEGF injections have shown benefit in treatment of CNVM. There is very little information about benefit of IVZ in CSCR related CNVM.* Conclusion.* IVZ is effective in regression of CSCR related CNVM and is associated with better macular anatomy and improved visual function.

## 1. Case Report

48-year-old male presented with history of gradual blurring of vision in right eye (OD) for the past 3 years. He was a known case of CSCR for which he had been managed conservatively with observation. Patient gave history of using tablet prednisolone for joint pain on and off over the past 4 to 5 years. Apart from the oral steroid prescriptions, his other orthopedic checkup records were not available. Patient denied any other systemic illness. He was not a smoker.

## 2. Examination

### 2.1. Right Eye (OD)

**Figure 1:**
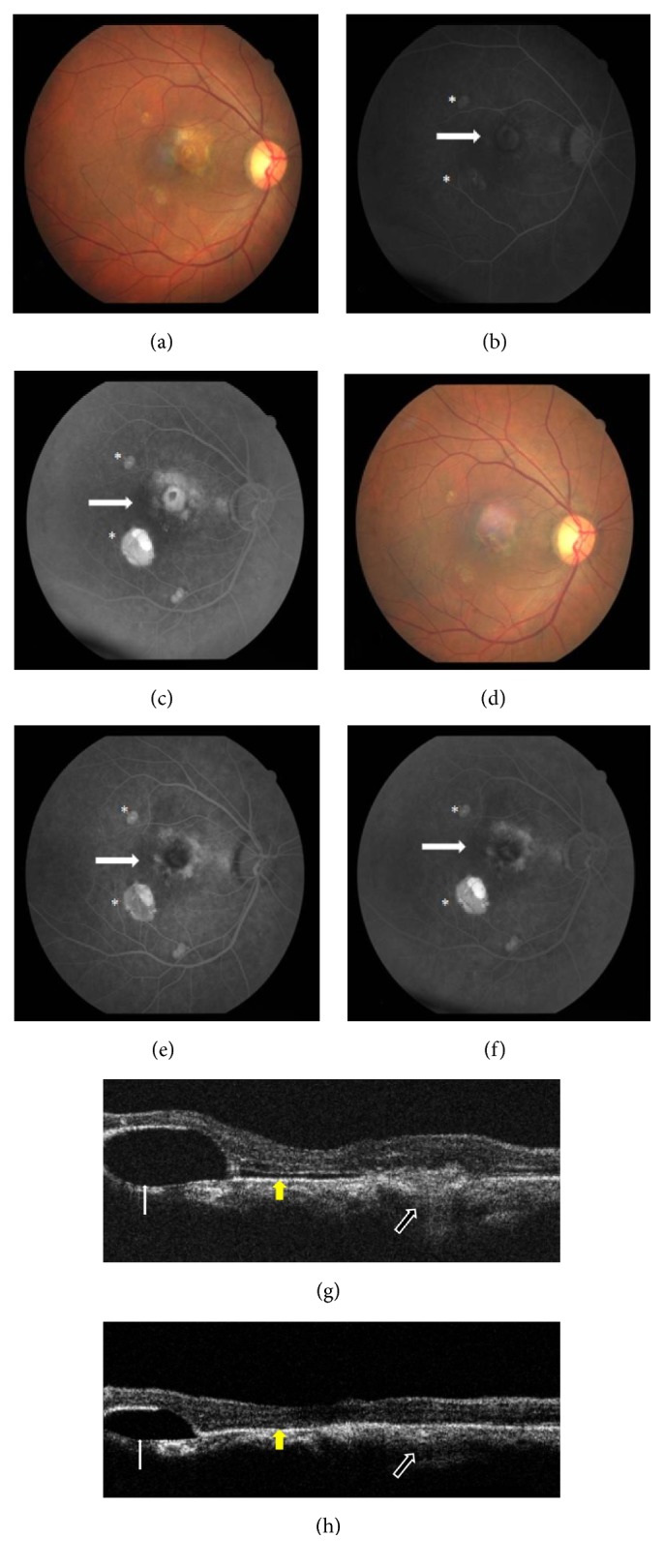
*Right eye pre- and posttreatment fundus images, FFA and OCT scans*. (a) Pretreatment color fundus image showing PED with CNVM. (b) and (c) Pretreatment FFA with 2 PEDs (asterix) and CNVM (white arrow). (d) Posttreatment color fundus image showing scarred CNVM with flattened PEDs. (e) and (f) Posttreatment FFA showing scarred CNVM with no leakage. (g) Pretreatment OCT demonstrating submacular CNVM complex (broad arrow with white borders), SRF (yellow arrow), and PED (narrow white arrow). (h) Posttreatment OCT demonstrating regressed CNVM complex (broad arrow with white borders), resolved SRF (yellow arrow), and reduced PED height (narrow white arrow).

Best corrected visual acuity (BCVA) was 3/60 and <N36. Anterior segment examination was normal on slit lamp biomicroscopy. Intraocular pressure (IOP) was 14 mmHg. Optic disc showed 0.5 : 1 cup : disc ratio with temporal pallor. Fundus examination revealed two Pigment Epithelial Detachments (PEDs) with Retinal Pigment Epithelium (RPE) alterations: superotemporal and inferotemporal to the fovea. There was a large partially scarred grayish yellow submacular membrane of about 1 disc diameter in size with small streak of subretinal hemorrhage suggestive of Choroidal Neovascular Membrane (CNVM) (Figure 1(a)). There were surrounding RPE alterations and atrophy. Peripheral retinal examination was normal.

### 2.2. Left Eye (OS)

**Figure 2:**
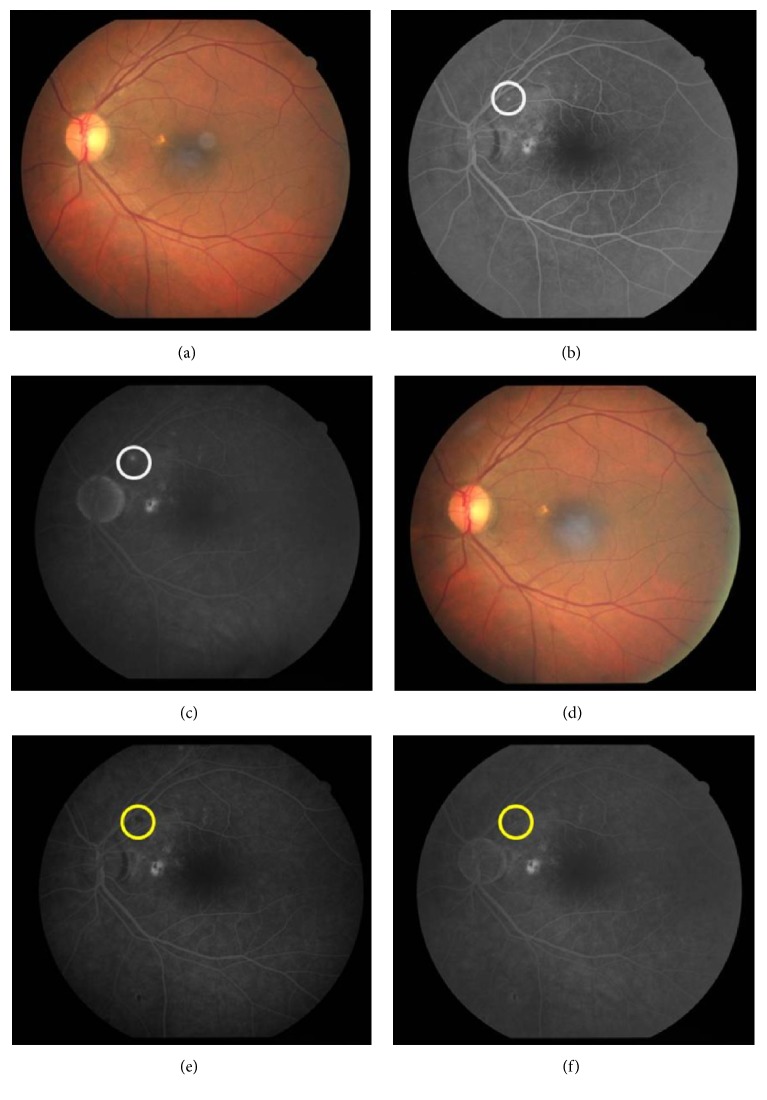
*Left eye pre- and posttreatment fundus images, FFA and OCT scans*. (a) Pretreatment color fundus image showing RPE alterations over papilla-macular bundle. (b) and (c) Pretreatment FFA with transmission defects and ink blot hyperfluorescence (white circle). (d) Posttreatment color fundus image showing RPE alterations suggestive of resolved CSCR. (e) and (f) Posttreatment FFA showing healed CSCR with no leakage (yellow circle).

BCVA was 6/9 and N6. Anterior segment examination was normal on slit lamp biomicroscopy. Intraocular pressure (IOP) was 16 mmHg. Optic disc examination revealed 0.4 : 1 cup : disc ratio. There were subtle retinal pigment epithelium (RPE) alterations over the papilla-macular bundle (PMB) (Figure 2(a)). There was a suspicious area superonasal to the fovea which had a hint of water mark on 78 D biomicroscopy. Features were suggestive of CSCR sequelae. Peripheral retina evaluation was normal.

## 3. Investigations

Fundus Fluorescein Angiography (FFA) in OD revealed features of classic CNVM with a scarred component (Figures 1(b) and 1(c)). There were areas of transmitted hyperfluorescence and blocked fluorescence around the CNVM. 2 PEDs were noted of which the inferotemporal one was large. FFA in OS revealed transmission window defects over the papilla-macular bundle and superonasally (Figures 2(b) and 2(c)). There was also a small PED along the PMB. A small ink blot pattern of hyperfluorescence was seen along the superotemporal arcade. These features were suggestive of chronic CSCR sequelae with focus of activity.

Optical coherence tomography (OCT) (Stratus OCT, Carl Zeiss Meditec AG, Germany) was done using Raster line scanning protocol. OD revealed a blunted foveal contour, cystoid changes in the macula along with a shallow layer of SRF, and submacular CNVM complex (Figure 1(g)). It also demonstrated a large tense PED inferotemporally and a small PED superotemporally. OS revealed presence of a normal foveal contour and normal central retinal thickness. There were RPE alterations along the PMB nasally and superonasal to the fovea along with a small PED on the PMB. SRF was observed superonasal to the fovea along the superotemporal arcade vessel corresponding to the area of ink blot leakage on FFA, suggestive of active CSCR and its sequelae. There was no evidence of CNVM. Based on these findings diagnosis of OD: Chronic CSCR associated CNVM and OS: Chronic CSCR was made.

## 4. Treatment

Intravitreal Ziv-Aflibercept (Zaltrap, Regeneron pharmaceuticals Inc, USA) injection (0.05 ml, 1.25 mg) was given in OD. 3 injections were given on monthly intervals.

In OS angiography guided focal laser to the CSCR leak was done. Off label use of Ziv-Aflibercept was explained to the patient and informed consent was obtained. Institutional review board clearance was taken for treatment and procedures were performed according to tenets of the Declaration of Helsinki.

## 5. Results

**Figure 3:**
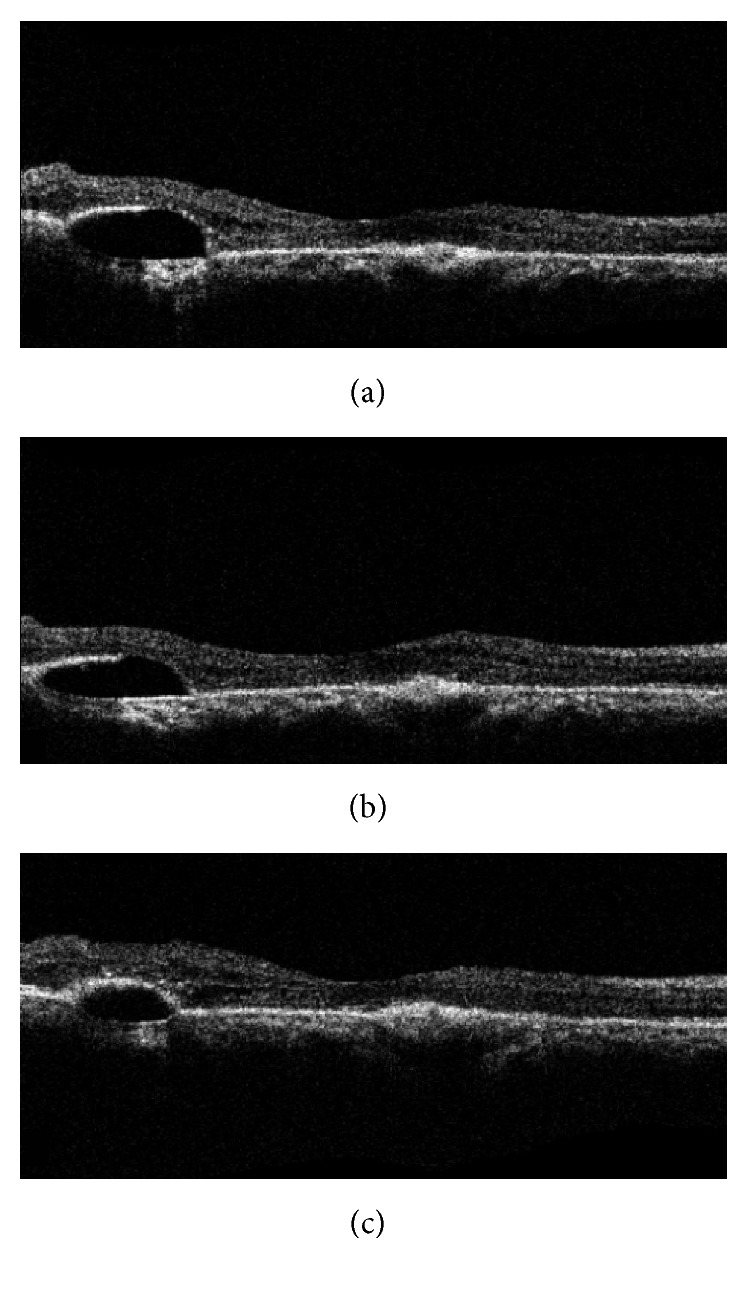
*Right eye posttreatment sequential OCT scans demonstrating reduction in PED height and regressing CNVM*. (a) After 1st injection, (b) after 2nd injection, and (c) after 3rd injection.

With each intravitreal injection, there was improvement in BCVA and regression of CNVM (Table 1). OCT revealed regression, in form of decrease in central macular thickness, reduced subretinal fluid, and increased scar component (Figures 3(a)–3(c)). Notably the infero-temporal PED height had also reduced and superotemporal PED had flattened (Figure 1(h)). OS revealed a dry looking macula with residual RPE alterations as noted earlier. Height of the PED had also reduced. These features were suggestive of resolved CSCR. At last follow-up (6 months), BCVA in OD had improved to 6/12, N12 and OS was maintained at 6/9, N6 (Table 1). FFA demonstrated a scarred CNVM with no leakage in OD (Figure 1(f)). OS revealed healed CSCR without any active leaks (Figure 2(f)).

## 6. Discussion

CSCR is a localized serous detachment of neurosensory retina occurring most commonly in young adults. CSCR is postulated to be caused primarily by abnormal choroidal vascular leading to overlying RPE dysfunction [1]. Other eye of the patient may also demonstrate choroidal and RPE abnormalities [2]. Chronic CSCR causes persistent RPE dysfunction and subsequent RPE atrophy leading to permanent visual dysfunction. Cystoid macular edema, subretinal lipid deposits, and CNVM are some of the long term sequelae of this disease.

CSCR resolves spontaneously in many patients. Focal laser photocoagulation (LP) and photodynamic therapy (PDT) with verteporfin have been used to treat CSCR. LP and PDT treated eyes may incur thermal injury and permanent RPE damage and develop CNVM [3].

Treatment with intravitreal anti-vascular endothelial growth factors (VEGF) has demonstrated early resolution of CSCR [4, 5]. Choroidal ischemia as demonstrated on ICG may lead to an increase in VEGF levels. VEGF increases vascular permeability leading to choroidal hyperpermeability which is thought to be the underlying mechanism of CSCR. With the use of Anti-VEGF, choroidal vascular permeability may be reduced leading to early resorption of SRF.

Various Anti-VEGF have been used in treatment of CSCR like bevacizumab, ranibizumab, and aflibercept. VEGF Trap (aflibercept) not only binds multiple isoforms of VEGF-A but also inhibits the activation of VEGFR1 and VEGFR2. VEGF Trap binds to VEGF-A faster and with higher affinity than bevacizumab and ranibizumab [6].

Aflibercept (VEGF trap Eye: Eylea, Regeneron Pharmaceuticals, Tarrytown, New York, USA) is seldom used in the developing countries due to its high cost and lack of availability. We used Ziv-Aflibercept instead of Eylea because of nonaffordability of our patient. Ziv-aflibercept in dosage of 1.25 mg/0.05 ml has been demonstrated to be safe for intravitreal usage [7]. Apart from diabetic macular edema and AMD related CNVM, it has been used for treatment of polypoidal choroidal vasculopathy (PCV) and RPE detachment. Aflibercept (Eylea) has been used to treat CNVM secondary to laser/PDT in CSCR [8]. PDT has been used in treatment of CNVM secondary to idiopathic CSCR.

Our case was a treatment naïve CSCR related CNVM. BCVA improved from 2/60 before treatment to 6/12 after 3 injections of Ziv-Aflibercept. OCT demonstrated resolution of CNVM with resorption of SRF. Height of PED also reduced on subsequent follow-ups. There were no ocular or systemic complications associated with the drug usage. OS showed chronic CSCR with diffuse RPE dysfunction. Presence of RPE abnormalities in contralateral asymptomatic eye has been mentioned in literature and has been demonstrated on ERG and ICG [2]. Contra lateral eye in cases of unilateral CSCR need to be screened at regular intervals along with the affected eye as there is risk of subclinical CSCR or development of CSCR in the future.

## 7. Conclusion

Intravitreal injection of Ziv-Aflibercept is a safe and effective treatment option for treatment of chronic untreated CSCR related CNVM.

## Figures and Tables

**Table 1:** Visual acuity and macular thickness.

	BCVA^*∗*^ OD^†^	CMT^‡^ on OCT^*∗∗*^ (micron)
Pretreatment	3/60, <N36	289
After 1st INJ^††^	6/36P, N24	230
After 2nd INJ^††^	6/18, N18	216
After 3rd INJ^††^	6/12P, N12	210
At 6 months	6/12, N12	208

^*∗*^BCVA: best corrected visual acuity; ^†^OD: right eye; ^‡^CMT: central macular thickness; ^*∗∗*^OCT: Optical Coherence Tomography; ^††^INJ: injection.
